# Usable and Privacy-Enhanced Telepresence Robots for Older Adults Aging in Place

**DOI:** 10.1093/geroni/igaa057.635

**Published:** 2020-12-16

**Authors:** Xian Wu, Jenay Beer

**Affiliations:** 1 University of Georgia, Athen, Georgia, United States; 2 Institute of Gerontology, Athens, Georgia, United States

## Abstract

Aging population is growing rapidly in the United States and people are living longer. Maintaining health and wellness while aging-in-place is crucial for older adults. Telepresence technology is beneficial for this target population to stay socially connected, as well as utilize telehealth and telemedicine services. However, such technology was not specifically designed for older adults. For older users to adopt telepresence, it is important to ensure that they do not experience adoption barriers, such as issues with usability and privacy. This research used a user-centered evaluation to design, develop, and test telepresence user interfaces (UI). Thirty older adults (aged 60+) participated in a within-subjects evaluation of two telepresence UIs: 1) the controlled condition - a generic UI, called Presence, based on currently available telepresence systems; and 2) the experiment condition - an enhanced custom telepresence UI that was designed follow human factors and design principles for older adults, named InTouch. Participants tested both UIs in a virtual home environment developed in Unity. Qualitative and quantitative results suggest that older adults perceived the experiment condition – InTouch, to be more usable and private – and our older users provided insight on which usability and privacy features were critical for them. By investigating the design of telepresence for older users, and applying those findings to design recommendations, we aim to improve the ease of use and privacy level of telepresence– not only for our target users but for all users who wish to enhance social connectedness and utilize telehealth.

